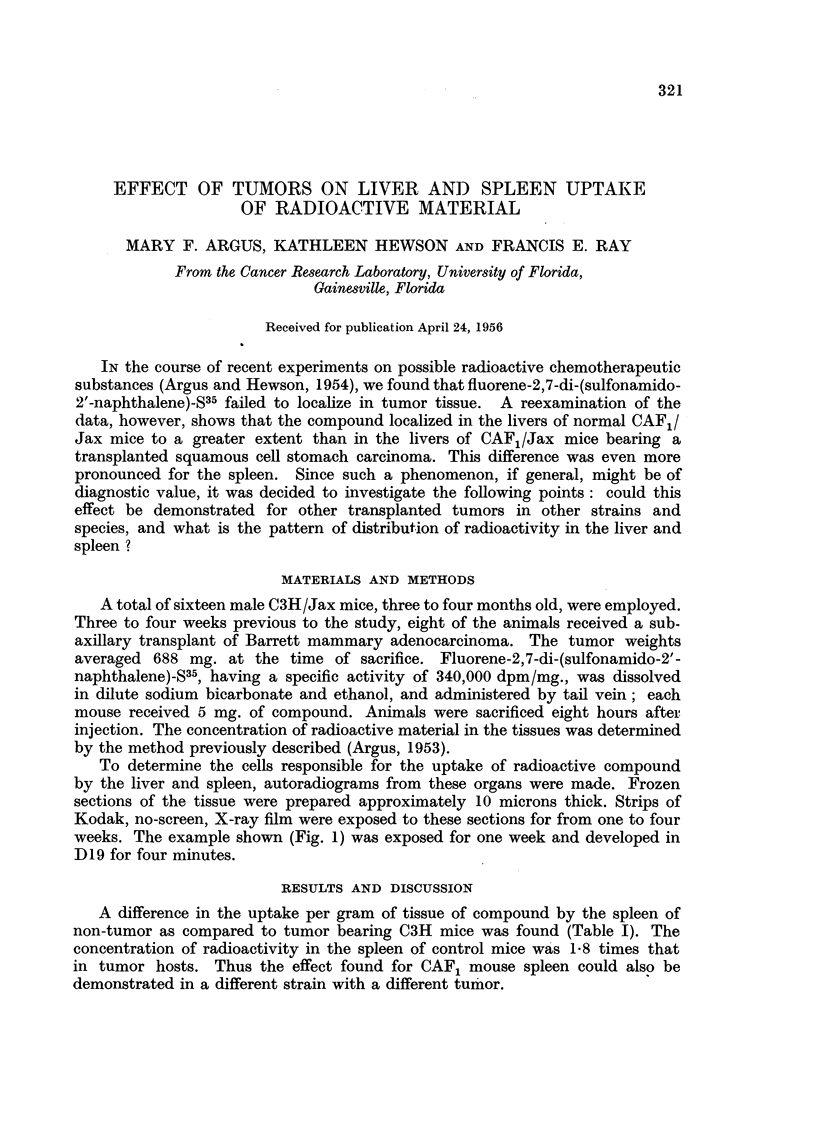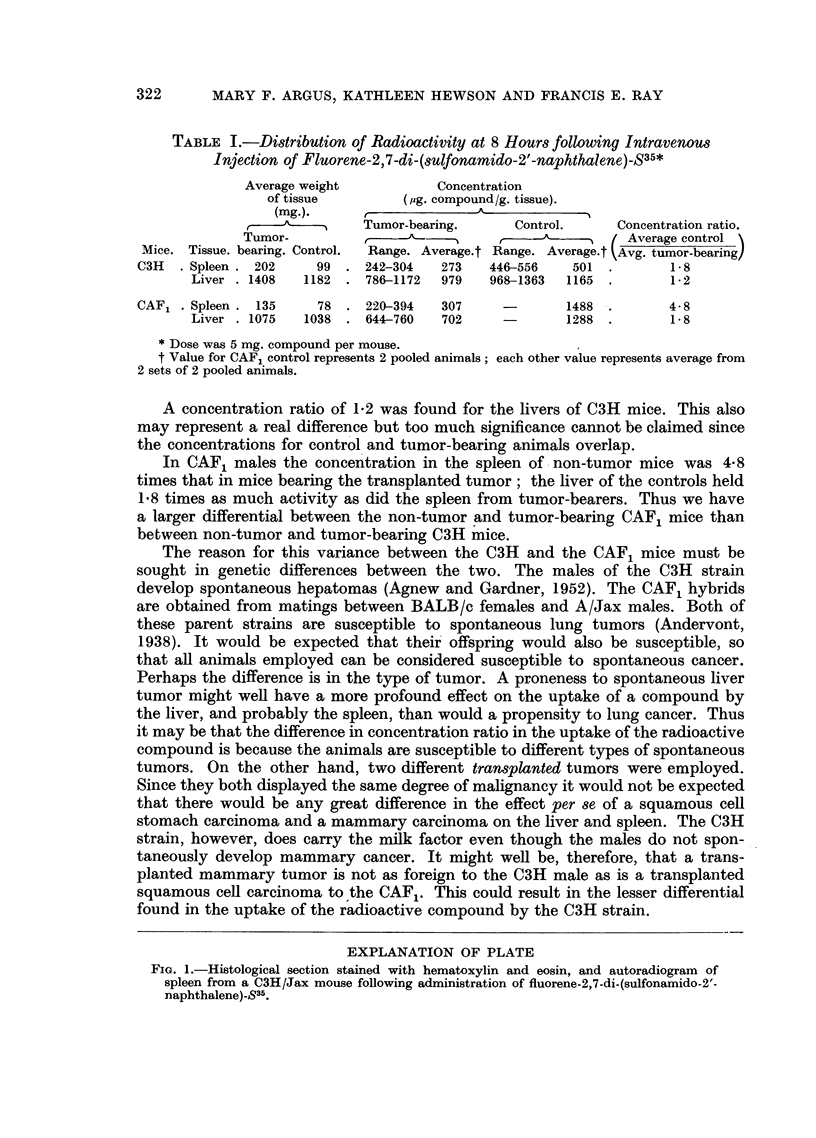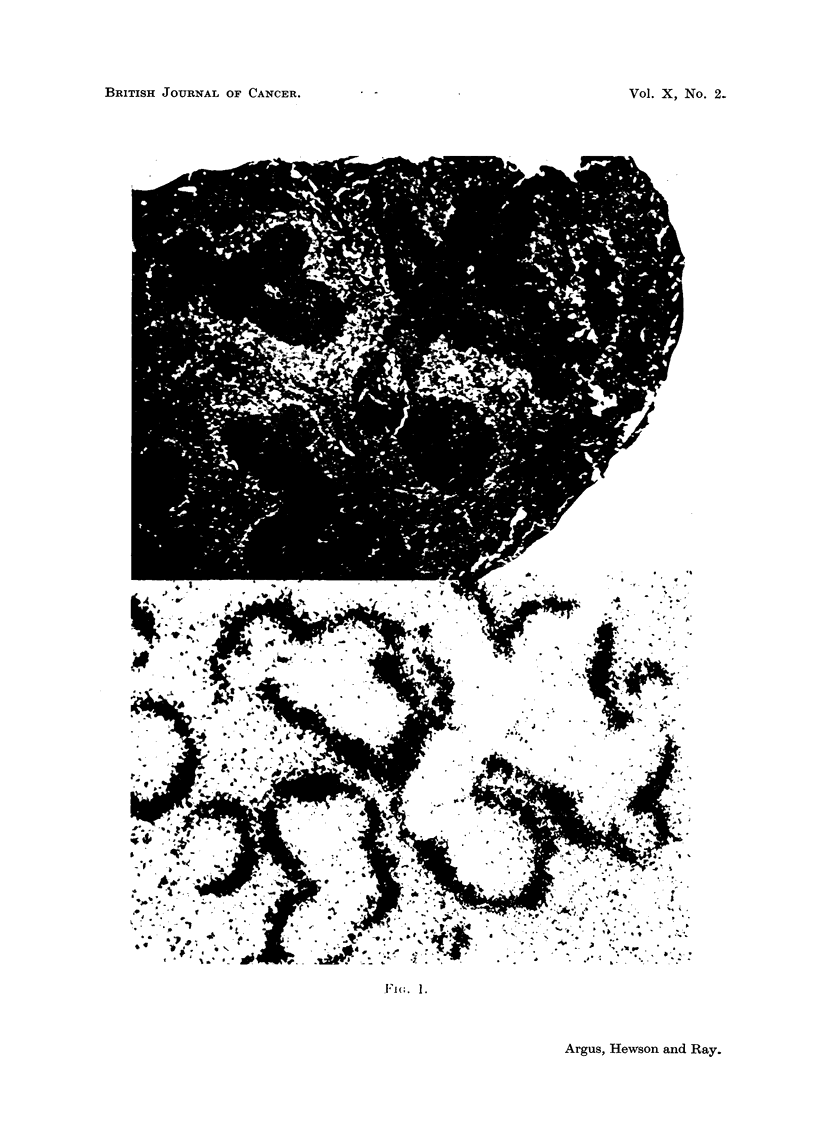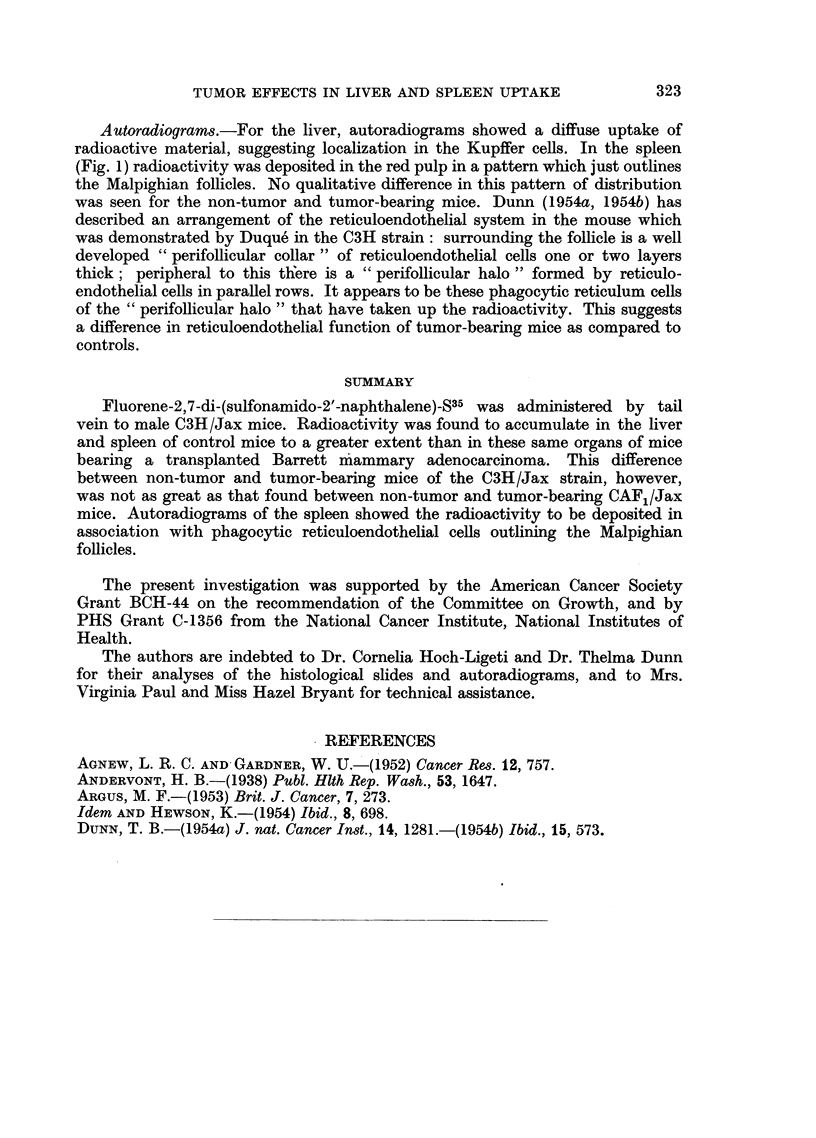# Effect of Tumors on Liver and Spleen Uptake of Radioactive Material

**DOI:** 10.1038/bjc.1956.38

**Published:** 1956-06

**Authors:** Mary F. Argus, Kathleen Hewson, Francis E. Ray

## Abstract

**Images:**


### EFFECT OF TUMORS ON LIVER AND SPLEEN UPTAKE OF RADIOACTIVE MATERIAL

MARY F. ARGUS, KATHLEEN HEWSON AND FRANCIS E. RAY

*From the Cancer Research Laboratory, University of Florida, Gainesville, Florida*

Received for publication April 24, 1956

IN the course of recent experiments on possible radioactive chemotherapeutic substances (Argus and Hewson, 1954), we found that fluorene-2,7-di-(sulfonamido-2′-naphthalene)-$S^{35}$ failed to localize in tumor tissue. A reexamination of the data, however, shows that the compound localized in the livers of normal $CAF_1$/Jax mice to a greater extent than in the livers of $CAF_1$/Jax mice bearing a transplanted squamous cell stomach carcinoma. This difference was even more pronounced for the spleen. Since such a phenomenon, if general, might be of diagnostic value, it was decided to investigate the following points : could this effect be demonstrated for other transplanted tumors in other strains and species, and what is the pattern of distribution of radioactivity in the liver and spleen ?

### MATERIALS AND METHODS

A total of sixteen male C3H/Jax mice, three to four months old, were employed. Three to four weeks previous to the study, eight of the animals received a sub-axillary transplant of Barrett mammary adenocarcinoma. The tumor weights averaged 688 mg. at the time of sacrifice. Fluorene-2,7-di-(sulfonamido-2′-naphthalene)-$S^{35}$, having a specific activity of 340,000 dpm/mg., was dissolved in dilute sodium bicarbonate and ethanol, and administered by tail vein ; each mouse received 5 mg. of compound. Animals were sacrificed eight hours after injection. The concentration of radioactive material in the tissues was determined by the method previously described (Argus, 1953).

To determine the cells responsible for the uptake of radioactive compound by the liver and spleen, autoradiograms from these organs were made. Frozen sections of the tissue were prepared approximately 10 microns thick. Strips of Kodak, no-screen, X-ray film were exposed to these sections for from one to four weeks. The example shown (Fig. 1) was exposed for one week and developed in D19 for four minutes.

### RESULTS AND DISCUSSION

A difference in the uptake per gram of tissue of compound by the spleen of non-tumor as compared to tumor bearing C3H mice was found (Table I). The concentration of radioactivity in the spleen of control mice was 1·8 times that in tumor hosts. Thus the effect found for $CAF_1$ mouse spleen could also be demonstrated in a different strain with a different tumor.

TABLE I.—*Distribution of Radioactivity at 8 Hours following Intravenous Injection of Fluorene-2,7-di-(sulfonamido-2'-naphthalene)-$S^{35}$**

| Mice. | Tissue. | Average weight of tissue (mg.). Tumor-bearing. | Control. | Concentration ($\mu$g. compound/g. tissue). Tumor-bearing. Range. | Average.† | Control. Range. | Average.† | Concentration ratio. (Average control / Avg. tumor-bearing) |
|---|---|---|---|---|---|---|---|---|
| C3H | Spleen | 202 | 99 | 242–304 | 273 | 446–556 | 501 | 1·8 |
| | Liver | 1408 | 1182 | 786–1172 | 979 | 968–1363 | 1165 | 1·2 |
| $CAF_1$ | Spleen | 135 | 78 | 220–394 | 307 | — | 1488 | 4·8 |
| | Liver | 1075 | 1038 | 644–760 | 702 | — | 1288 | 1·8 |

* Dose was 5 mg. compound per mouse.

† Value for $CAF_1$ control represents 2 pooled animals ; each other value represents average from 2 sets of 2 pooled animals.

A concentration ratio of 1·2 was found for the livers of C3H mice. This also may represent a real difference but too much significance cannot be claimed since the concentrations for control and tumor-bearing animals overlap.

In $CAF_1$ males the concentration in the spleen of non-tumor mice was 4·8 times that in mice bearing the transplanted tumor ; the liver of the controls held 1·8 times as much activity as did the spleen from tumor-bearers. Thus we have a larger differential between the non-tumor and tumor-bearing $CAF_1$ mice than between non-tumor and tumor-bearing C3H mice.

The reason for this variance between the C3H and the $CAF_1$ mice must be sought in genetic differences between the two. The males of the C3H strain develop spontaneous hepatomas (Agnew and Gardner, 1952). The $CAF_1$ hybrids are obtained from matings between BALB/c females and A/Jax males. Both of these parent strains are susceptible to spontaneous lung tumors (Andervont, 1938). It would be expected that their offspring would also be susceptible, so that all animals employed can be considered susceptible to spontaneous cancer. Perhaps the difference is in the type of tumor. A proneness to spontaneous liver tumor might well have a more profound effect on the uptake of a compound by the liver, and probably the spleen, than would a propensity to lung cancer. Thus it may be that the difference in concentration ratio in the uptake of the radioactive compound is because the animals are susceptible to different types of spontaneous tumors. On the other hand, two different *transplanted* tumors were employed. Since they both displayed the same degree of malignancy it would not be expected that there would be any great difference in the effect *per se* of a squamous cell stomach carcinoma and a mammary carcinoma on the liver and spleen. The C3H strain, however, does carry the milk factor even though the males do not spontaneously develop mammary cancer. It might well be, therefore, that a transplanted mammary tumor is not as foreign to the C3H male as is a transplanted squamous cell carcinoma to the $CAF_1$. This could result in the lesser differential found in the uptake of the radioactive compound by the C3H strain.

---

EXPLANATION OF PLATE

FIG. 1.—Histological section stained with hematoxylin and eosin, and autoradiogram of spleen from a C3H/Jax mouse following administration of fluorene-2,7-di-(sulfonamido-2'-naphthalene)-$S^{35}$.

FIG. 1.

*Autoradiograms.*—For the liver, autoradiograms showed a diffuse uptake of radioactive material, suggesting localization in the Kupffer cells. In the spleen (Fig. 1) radioactivity was deposited in the red pulp in a pattern which just outlines the Malpighian follicles. No qualitative difference in this pattern of distribution was seen for the non-tumor and tumor-bearing mice. Dunn (1954*a*, 1954*b*) has described an arrangement of the reticuloendothelial system in the mouse which was demonstrated by Duqué in the C3H strain : surrounding the follicle is a well developed " perifollicular collar " of reticuloendothelial cells one or two layers thick ; peripheral to this there is a " perifollicular halo " formed by reticuloendothelial cells in parallel rows. It appears to be these phagocytic reticulum cells of the " perifollicular halo " that have taken up the radioactivity. This suggests a difference in reticuloendothelial function of tumor-bearing mice as compared to controls.

SUMMARY

Fluorene-2,7-di-(sulfonamido-2′-naphthalene)-$S^{35}$ was administered by tail vein to male C3H/Jax mice. Radioactivity was found to accumulate in the liver and spleen of control mice to a greater extent than in these same organs of mice bearing a transplanted Barrett mammary adenocarcinoma. This difference between non-tumor and tumor-bearing mice of the C3H/Jax strain, however, was not as great as that found between non-tumor and tumor-bearing $CAF_1$/Jax mice. Autoradiograms of the spleen showed the radioactivity to be deposited in association with phagocytic reticuloendothelial cells outlining the Malpighian follicles.

The present investigation was supported by the American Cancer Society Grant BCH-44 on the recommendation of the Committee on Growth, and by PHS Grant C-1356 from the National Cancer Institute, National Institutes of Health.

The authors are indebted to Dr. Cornelia Hoch-Ligeti and Dr. Thelma Dunn for their analyses of the histological slides and autoradiograms, and to Mrs. Virginia Paul and Miss Hazel Bryant for technical assistance.

### REFERENCES

AGNEW, L. R. C. AND GARDNER, W. U.—(1952) *Cancer Res.* **12**, 757.
ANDERVONT, H. B.—(1938) *Publ. Hlth Rep. Wash.*, **53**, 1647.
ARGUS, M. F.—(1953) *Brit. J. Cancer*, **7**, 273.
*Idem* AND HEWSON, K.—(1954) *Ibid.*, **8**, 698.
DUNN, T. B.—(1954*a*) *J. nat. Cancer Inst.*, **14**, 1281.—(1954*b*) *Ibid.*, **15**, 573.